# Treatment of Hypertrophy of the Prostate at the Northampton General Infirmary

**Published:** 1895-04-27

**Authors:** 


### TREATMENT OF HYPERTROPHY OF PROSTATE AT THE NORTHAMPTON GENERAL INFIRMARY.

(Communicated.)

Judging from published reports, there are few diseases in which the treatment is so varying at different hospitals as in these cases. Thus one reads of numerous cases of castration, or of removal of portions of the prostate by supra-pubic incision at some hospitals, in which one cannot but think that either the early treatment of these cases has been neglected, or that some bold surgeon is anxious to try the latest remedy, however severe, that he may be the first to chronicle his successes in a new line. It is however in the early treatment of these cases that most good may be effected. A patient comes complaining of a dribbling stream of urine; of being compelled to pass it very frequently; of restless nights caused through this necessity, and of great mental trouble and consequent distress. These symptoms have been going on for some time, and are gradually getting worse, and on examination (should such be deemed necessary) some enlargement of the prostate will be found. Yet there can be no great amount of residual urine, and in such early cases the passage of the catheter would be inexcusable. If the patient can be kept quiet in bed for a few days it may facilitate the cure, but in the large majority small doses of strychnine given regularly will effect a marked improvement. Men have come with the above symptoms, and after a course of strychnine they have improved wonderfully, have rested well at night, and declared they felt new persons; in fact, if one may judge of such cases from their non-return in the event of a relapse, some must have recovered altogether, whilst many have had a long interval of ease and comfort. If strychnine should not benefit them another drug—ergot—may be tried. What is the rationale of the treatment: why in one case strychnine should do good, whilst in another ergot is more beneficial, it is difficult to say; but it is only one of the peculiarities met with so frequently in our treatment of all diseases.

Passing on to the next stage, where there is some residual urine left which causes trouble; rest in bed, with catheterism twice or three times a day, and washing out the bladder with boric acid will do much good in association with the above drugs—strychnine or ergot—in mild cases. It is not the practice here to condemn a man to catheter life too quickly, and many of these cases will get better and practically well for some time under the above treatment, but, of course, in more severe cases the catheter and regular washing out of the bladder with some antiseptic (usually boric acid is used) will be required. As to the much vaunted treatment in severe cases of internal doses of boric acid or salol, it is impossible to give that great praise which some surgeons have done. Here it has been of very little use, though it has been often tried in these and analogous cases. Whether the cases have been too severe, or whether it was not administered properly, the fact remains that very little benefit has been observed from such treatment.

When catheter life is failing; when the instrument has to be passed so frequently that there is little rest or comfort, and cystitis and other evils are added, much relief has been given by Thompson's operation of opening the bladder through the perineum, and draining it. The operation is most simple and most easily performed, although of late it has given way to more fashionable operative measures, yet it is so sound in its principles and so effective in its results that it is bound to survive. One man, some years ago, was a most pitiable object, passing water day and night every quarter of an hour, with frightful straining and all the other horrors of this disease; yet directly after the operation the effect was most marvellous. He slept, took his meals again, and picked up so much that he left the infirmary comparatively well, and continued so certainly for some time till we at last lost sight of him.

As to the other operations for the relief of this disease—castration and removal of portions of the prostate by supra-pubic incision—it will suffice to say that they are still on their trial, and since they have been brought forward no suitable case for such treatment has presented itself here. By incessant care in the early treatment of these cases, first without, then with, well-regulated catheter life, and by the adoption of Thompson's operation in the more severe cases, it will be rare indeed for such severe measures to be called for as to justify recourse to the larger operations.

